# Comparative Analysis of Surgical Methods for Distal, Mid-, and Proximal Shaft Hypospadias in Young Males: A Prospective Study on Postoperative Outcomes

**DOI:** 10.3390/medicina60111903

**Published:** 2024-11-20

**Authors:** Almira Zharkimbayeva, Maratbek Aubakirov, Vasily Lozovoy, Madina Madiyeva, Samatbek Abdrakhmanov, Azat Dyussembayev

**Affiliations:** 1 Department of Traumatology and Pediatric Surgery, Semey Medical University, Semey 071400, Kazakhstan; almira.zharkimbayeva@smu.edu.kz (A.Z.); maratbek.aubakirov@smu.edu.kz (M.A.);; 2 Department of Pediatric Surgery, Astana Medical University, Astana 010000, Kazakhstan; v.m.loz@mail.ru; 3 Department of Radiology, Semey Medical University, Semey 071400, Kazakhstan; 4 Department of Surgery, Semey Medical University, Semey 071400, Kazakhstan; samatbek.abdrakhmanov@smu.edu.kz

**Keywords:** hypospadias, urethroplasty, surgical treatment, complications, fistulas

## Abstract

*Background and Objectives:* The purpose of this study is to report on the results of hypospadias surgery in boys using newly developed methods compared with traditional methods of urethroplasty. *Materials and Methods:* A total of 136 patients were divided into two groups. Fifty patients with coronal, subcoronal, and distal penile types of hypospadias were allocated to Group I. These patients were treated with a new method of SMU I and urethroplasty according to the Snodgrass procedure and MAGPI. Group II consisted of 86 patients with mid- and proximal shaft penile types of hypospadias who underwent urethroplasty using the new SMU II method and urethroplasty according to the Snodgraft and Bracka procedures. The outcomes were assessed according to the level of postoperative wound healing and the presence of complications. *Results:* The mean age of the patients was 41 to 60 months. After urethroplasty using the SMU I and SMU II methods, recovery was noted in 92.9% of the patients, and repeated surgery was performed in 7.1%. In the patients undergoing operations utilizing the SMU I and SMU II methods, neomeatus dystopia was not detected and postoperative fistulas were observed in 16.7% of incidence. With the standard urethroplasty method, the rate of complications was higher (*p* < 0.05). Even though the duration of surgery in the group undergoing a procedure using the standard urethroplasty method was lower (*p* < 0.001), the frequency of primary wound healing was higher in the SMU I and SMU II groups (*p* < 0.001). *Conclusions:* The proposed new methods of distal and shaft hypospadias surgery, in comparison with traditional surgical techniques, have the following advantages: good cosmetic appearance of the penis, complete straightening of the shaft, and right outflow of urine through the slit-like neomeatus at the apex of the glans penis.

## 1. Introduction

Hypospadias occurs as a result of the abnormal or incomplete formation of the urethra in the first weeks of embryonic development [1]. The surgical method of treating hypospadias is aimed at restoring penis function and anatomy. The main objective of the treatment is to achieve a straight, aesthetically normal penis with a correctly positioned slit-like urethral meatus [2,3]. One of the important aspects in treating hypospadias is the patient’s age.

Previously, surgical treatment of hypospadias was recommended from the age of six months or from the age of one year. Currently, according to the recommendations of the American Academy of Pediatrics and European experts, hypospadias should be treated at the age of 6 to 12 months [4]. The average age at the time of the first operation using the urethroplasty method developed by us ranged from 10 months to 11 years. We have studied the influence of other factors on the final result of hypospadias surgical treatment. It was revealed that 31% of the complications after operations using the Duckett procedure, Mathieu-IP technique, tubularized incised plate (TIP) urethroplasty, and MAGPI (meatal advancement and glanuloplasty procedure) were influenced by the following factors: hypospadias severity (preoperative localization of passages and the presence of ventral curvature), the choice of correction method, and the surgeon’s experience in the surgical intervention technique. In many countries, surgeons apply various approaches to the assessment and correction of ventral curvature of the penile shaft [5]. When using TIP urethroplasty, complications such as urethrocutaneous fistulas, penile glans divergence, and meatal canal stenosis have been observed. The authors recommended long-term follow-up after surgery due to the relatively high rate of complications that occur during the first year post operation [6,7]. Thus, the choice of surgical treatment depends on the surgeon’s experience, the hypospadias type, and the degree of ventral curvature [8,9].

An analysis of the current data on the surgical treatment of hypospadias reveals the use of various urethroplasty techniques and methods by urologists. The urethroplasty method for treating various types of hypospadias in children developed by us has shown good results. Compliance with criteria, such as the correct choice of glanduloplasty method (the urethra should not be unacceptably deep in the spongy tissue), use of optical devices and rapidly absorbable suture material, protection of the suture line with darthos fascia, moderate compression of the penis during surgery, and immobilization in the postoperative period, reduce the risk of developing short- and long-term complications [10]. The purpose of this study was to evaluate the advantages of the urethroplasty method developed by us in terms of improving results and cosmetic appearance compared to those of standard methods.

## 2. Materials and Methods

### 2.1. Subjects

In accordance with the purpose and objectives of this study, a research protocol was developed [11]. Group I included 26 patients who underwent urethroplasty using the newly developed method of SMU I (Semey Medical University I) and 24 patients who underwent surgical methods of urethroplasty according to Snodgrass and MAGPI. Group I was divided into subgroups: A1 and A2. These subgroups of patients displayed coronal, subcoronal, and distal penile types of hypospadias. Group II included 33 patients who underwent urethroplasty using the newly developed method of SMU II (Semey Medical University II) and 53 patients who underwent surgery using the Snodgraft and Bracka methods. Group II was also divided into subgroups: B1 and B2. These subgroups of patients displayed midshaft penile and proximal shaft penile types of hypospadias. The dataset was collected from 2014 to 2023. The average age of patients in Group I was 49 months (95% CI: 53.04–72.69, SD = 37.7). The average age of patients in Group II was 59 months (95% CI: 53.94–67.10, SD = 28.9). The criteria for inclusion in this study were as follows: patients with the distal and middle-stem parts and lower third of the trunk of the penis and patients aged from 1 year to 17 years and 11 months. The exclusion criteria were as follows: patients with posterior hypospadias (scrotal, perineal); false hermaphroditism; patients with a history of postoperative complications such as urethral stenosis, urethrocutaneous fistula, and neurethral diverticulum; patients with excision of the foreskin; and patients with congenital malformations of the upper urinary system. The samples were comparable in age (*p* = 0.332).

This study was approved by the Ethics Committee of Semey Medical University (Protocol No. 9 dated 21 June 2022). All patients underwent invasive and non-invasive examination methods, including anamnesis, objective status, biochemical blood analysis, and karyotype (sex chromatin) determination. All patients underwent cystoscopy and uroflowmetry.

### 2.2. Ultrasound Dopplerography

**Figure 1:**
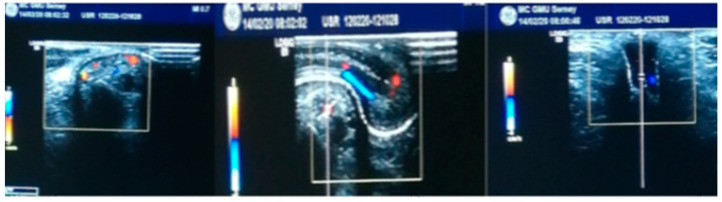
Duplex scanning of the vessels of the prepuce and penis. The arteries of the prepuce and penis skin are shown in red. The veins of the prepuce and penis skin are shown in blue.

Dopplerography of the vessels of the skin of the foreskin and penis was performed in order to select a vascularized flap with vessels of a larger diameter to create a urethral tube, as shown in Figure 1. At the same time, the average diameter of the dorsal artery was 0.14 cm on the right and 0.12 cm on the left. The linear blood flow velocity (LSC) was 23.9 cm/s and 20.4 cm/s (±0.01 cm), respectively.

### 2.3. Surgical Methods

**Figure 2:**
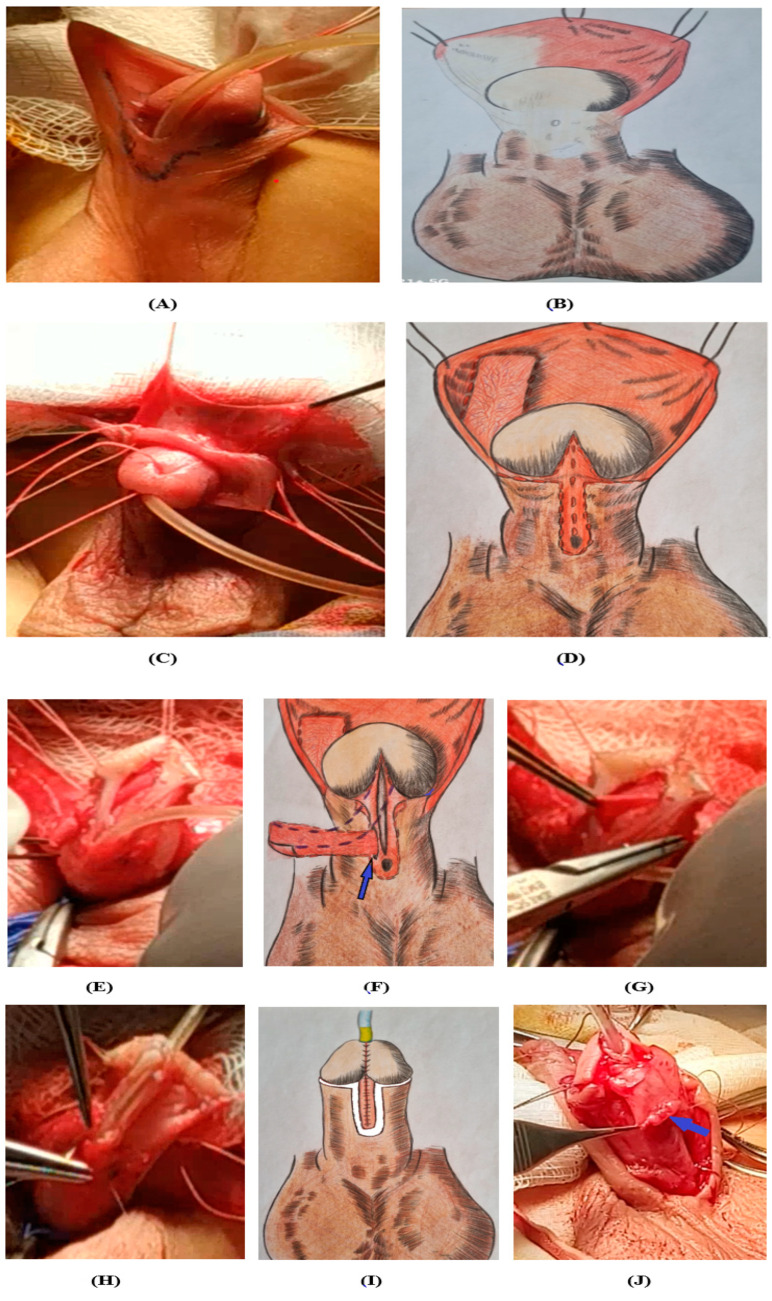

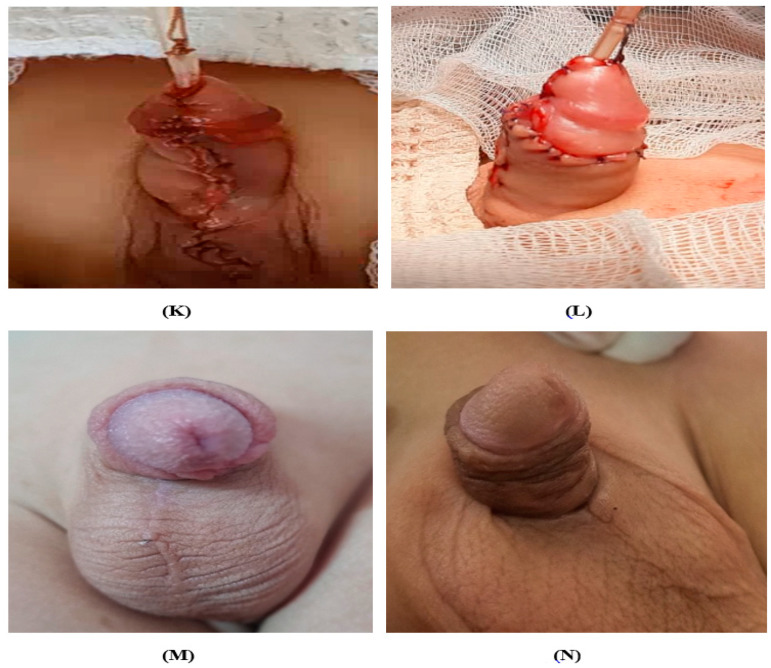
The stages of surgical intervention for the distal form of hypospadias: (**A**) The approximate lines of the incision and (**B**) scheme. (**C**) The skin of preputium and (**D**) cut lines are drawn in scheme. (**E**) The posterior urethral wall is dissected and (**F**) The fleshy membrane of the foreskin on the vascular pedicle (blue arrow) in scheme. (**G**) The inner wall of the dissected urethral site. (**H**) The creation of a urethral tube involves suturing the edges of the skin and (**I**) scheme. (**J**) The applying a protective suture (blue arrow) to the anterior wall of the neourethra. (**K**) Plastic surgery of the head and skin suturing with the creation of “raf” and (**L**) lateral view. (**M**) The appearance of the penis two months after urethroplasty by SMU I method and (**N**) six months after urethroplasty by SMU I method.

We have developed and applied a new method of surgery, SMU I, for subcoronal and distal shaft hypospadias [12], as illustrated in Figure 2 (methods of surgery in patients with subcoronal and distal shaft hypospadias). After applying the approximate lines of the incision, the skin was dissected. Stepping back from the coronary sulcus 0.5 cm along the perimeter of the penile shaft, a flap is cut out on the ventral surface of the skin of the penile shaft with the border of the dystopic external opening of the urethra, and continuing to the top of the glans, the skin of the flap is dissected along the midline (Figure 2A–D). A graft is cut out of the fleshy membrane of the foreskin on the vascular pedicle and moved through the created “window” at the inner wall of the dissected urethral site (Figure 2E–G). A graft on the feeding vascular pedicle is placed at the bottom of the urethral site, which is carefully fixed to the tissue of the glans and the tunica albuginea of the penile trunk. The anterior wall of the neourethra is formed at the top of the glans by suturing the edges of the dissected skin flap above the urethral catheter. The created urethra is covered with a protective layer of residual flaps of de-epithelialized foreskin, which is moved around the glans to the ventral surface (Figure 2H–J). Glanduloplasty proceeds as follows. The edges of the skin of the penile shaft are sutured. The Nelaton catheter is fixed to the head with a suture (Figure 2K,L). The results 2 months after surgical treatment are shown (Figure 2M,N).

**Figure 3:**
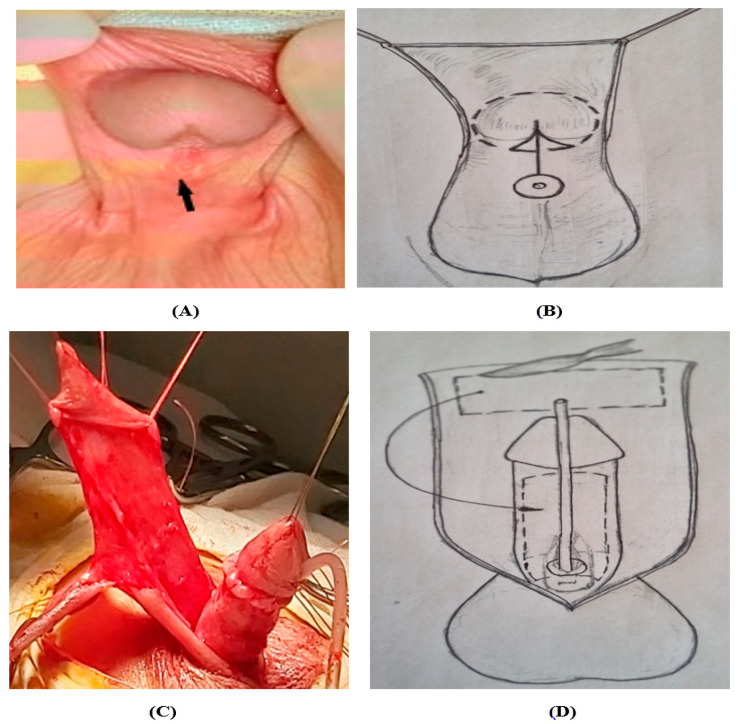

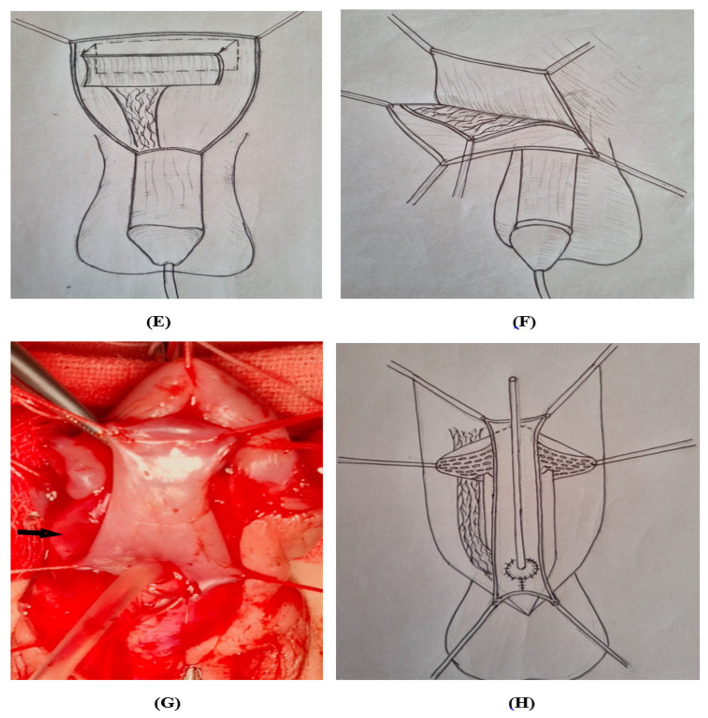

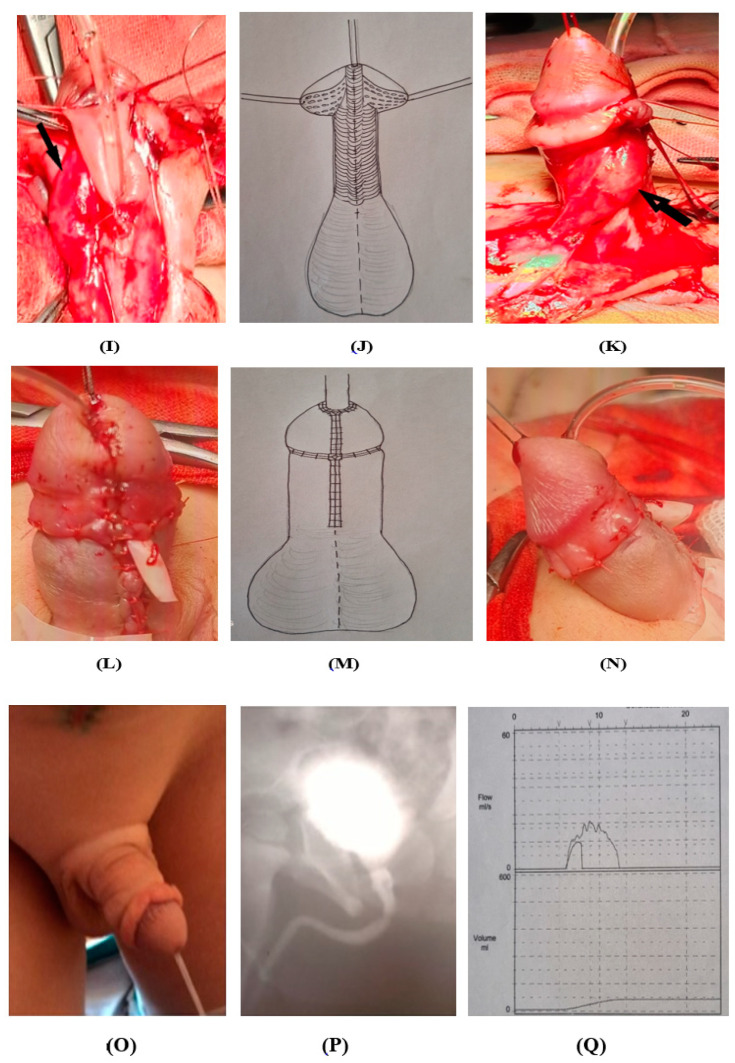
The stages of surgical intervention for midshaft and proximal shaft penile forms of hypospadias. (**A**) The meatus is located in the midshaft of the penis and (**B**) scheme. (**C**) Decutanization and excision of the chordee and (**D**) cut lines in scheme. (**E**) The mobilization of the graft on the vascular pedicle from the skin and fascia of the foreskin in frontal view in scheme and (**F**) in lateral view in scheme. (**G**) Moving it to the ventral surface of the penile shaft and (**H**) scheme. (**I**) Creating an anastomosis between the proximal part of the neourethra and the dystopian meatus and (**J**) scheme. (**K**) The graft on the vascular pedicle (black arrow). (**L**) Suturing of the urethral tube with an intradermal suture and (**M**) glanuloplasty in scheme. (**N**) Suturing the skin with the creation of “raf”. (**O**) The result of urethroplasty 3 months after surgical treatment with SMU II method. (**P**) uretrogramm after surgical treatment. (**Q**) Urofloogram 3 months after surgical treatment.

Surgical correction of midshaft and proximal forms of hypospadias is performed using the following traditional methods of surgery: the Snodgraft GTIP, Hodgson III, Duckett, Faizulin VIII, and Bracka procedures (in 1941, Humby first proposed the use of cheek mucosa). During general anesthesia, at the beginning of surgery, after applying a tourniquet to the base of the penis in order to determine the degree of ventral curvature, the Gittes–McLaughlin intraoperative technique was performed with 0.9% NaCl solution applied to the cavernous bodies. The angle of deformation and the angle of deviation of the top of the head from its normal position were measured by a protractor. Patients in the group with the midshaft form of hypospadias were operated on using the SMU II method [13], as shown in Figure 3. The method of surgical treatment of mid- and proximal shaft penile types of hypospadias includes the formation of a single integral (one complete) urethral tube from a transverse flap on the “feeding pedicle” of the inner layer of the foreskin and the fleshy membrane of the prepuce (on the dorsal surface) with a length equal to the distance from the apex of the glans to the external opening of the ectopic urethra and a width equal to 1/3 of its length (Figure 3A–D). After decutanization of the penile shaft and excision of embryonic adhesions and chordee, the mobilized graft with a vascular pedicle is moved by lowering without rotation to the ventral surface of the penis (Figure 3E–H), and the feeding flap is fixed to the fascia Buck with several sutures using a Vicryl 6/0 thread. The proximal part of the neourethra is formed up to 0.7 cm long by a bordering incision along the perimeter, and by stepping back 0.7 cm from the ectopic urethral orifice, and placing an anastomosis between the created skin collar and the dissected ends of the proximal part of the graft, a complete urethral tube is created by suturing the edges of the transplanted graft over the catheter (Figure 3I–K). The neourethral suture is covered along its entire length with a protective layer from the fascia of the ventral surface of the penis (this can be a fascial flap on a vascular pedicle) with a continuous suture. Glanduloplasty and suturing of the mobilized skin edges of the penile shaft is completed, with fixation of the Nelaton catheter (Figure 3L–N). The results 3 months after surgical treatment are shown (Figure 3O–Q).

### 2.4. Statistical Analysis

The frequencies were compared using either Pearson’s chi-square test or Fisher’s exact test (applied when expected cell values were five or less). The continuous variables were compared using the Mann–Whitney U-test due to their non-normal distribution, which was confirmed by means of the Shapiro–Wilk test. Statistical processing of the results was performed using the SPSS version 20 program (IBM Ireland Product Distribution Limited, Ireland).

## 3. Results

In the first stage, the incidence of various types of hypospadias and the average age of patients were studied. The compared groups had no statistically significant differences (*p* = 0.522, *p* = 0.573, *p* = 0.084, *p* = 0.641, respectively). The average age of the patients was 49 months (95% CI: 53.04–72.69), SD = 37.7, to 59 months (95% CI: 53.94–67.10), SD = 28.9 (Table 1 and Table 2).

When comparing the subgroups of Group I based on the types of hypospadias and clinical signs, as well as age, no statistically significant differences were found, as shown in Table 3 and Table 4. The average age of the patients was 48 months (95% CI: 45.29–60.64), SD = 19.0, and 51 months (95% CI: 46.08–65.00), SD = 22.4, respectively, as shown in Table 5.

When comparing subgroups of Group II based on the types of hypospadias and clinical signs, as well as age, no statistically significant differences were found, as shown in Table 6 and Table 7. The average age of the patients in subgroup B1 was 56 months (95% CI: 54.22–87.11), SD = 46.3, and in subgroup B2 was 60 months (95% CI: 54.10–71.44), SD = 31.4, as shown in Table 8.

Postoperative wound healing by primary tension in subgroup A1 (SMU I) was observed in all 26 patients, while in subgroup A2, healing by primary tension was observed in 17 patients; in the remaining seven cases, healing was by secondary tension. The postoperative complication of urethral fistula was observed in 12.1% (4) of the patients in subgroup B1 (SMU II) and 35.8% (19) of the patients in subgroup B2. This type of complication was absent in 87.9% (29) and 64.2% (34) of the patients, respectively. These results are presented in Table 9. Neomeatus dystopia was not observed in any of the patients in subgroup B1. Neomeatus dystopia was observed in 15.1% (8) of the patients in subgroup B2 (*p* = 0.016, *p* = 0.019). Postoperative wound healing by primary tension was observed in 97.0% (32) of the patients in subgroup B1 and in 69.8% (37) of the patients in subgroup B2. In subgroup B1, 3% (1), and in B2, 30.2% (16), of the patients exhibited healing by secondary tension (*p* = 0.002). According to the analysis, of the 50 patients from Group I who underwent an operative procedure, 80% (40) recovered and 20% (10) underwent repeated surgery. In subgroup A1, postoperative fistula was observed in 16.7% of the patients, and in subgroup A2, in patients who underwent Snodgrass and MAGPI surgery, fistula developed in 20.8% (5 patients) and neomeatus dystopia in 16.6% (4 patients) (*p* = 0.065; *p* = 0.030). According to the analysis, of the 86 patients from Group II who underwent an operative procedure, 61.6% (53) of the patients recovered and 38.4% (33) of the patients underwent repeated surgery. Of these, in subgroup B1, postoperative fistula was observed in 12.1% (4) of cases, whereas in subgroup B2, in those who underwent Snodgraft and Bracka surgery, fistula developed in 35.8% (19) of the patients and neomeatus dystopia in 15.1% (*p* = 0.016; *p* = 0.019). Fistula developed after urethroplasty at different time intervals from day 3 to day 10 of catheterization and was observed after the development of catheter urethritis at various points after neourethra stenting. In the postoperative period, the follow-up periods were as follows for investigation group Me-10 (Q1–Q3: 2–46) and comparison group Me-24 (Q1–Q3: 7–30). SMU I method: Me-15 (Q1–Q3: 2–60); SMU II method: Me-8 (Q1–Q3: 4–36). Subgroup A2 (Snodgrass and MAGPI): Me-24 (Q1–Q3: 14–35); B2 (Snodgraft and Bracka): Me-16 (Q1–Q3: 3–29).

## 4. Discussion

According to Duckett, distal forms account for 50% of hypospadias, medium for 30%, and proximal for 20% [14]. In our study, according to retrospective data, primary hypospadias amounted to 175 (57.6%) cases: the distal form was present in 68 patients (22.3%), average in 59 (19.3%), proximal in 40 (13.1%), and hypospadias without hypospadias in 8 (2.6%) [9].

Hypospadias, as a rule, is an isolated anomaly, but it can represent one of the features of more than 200 different syndromes and be accompanied by concomitant abnormalities of scrotal organ development [15,16]. Associated urinary tract malformations are the most common in proximal hypospadias. In the work described by the authors, the use of a two-dimensional ultrasound in combination with a three-dimensional ultrasound showed a tendency for higher informativeness compared with only two-dimensional investigations for the diagnosis of hypospadias in a fetus [17].

The generally accepted sonographic criteria for hypospadias diagnosis are a more rounded shape of the glans penis, short trunk of the penis, ventral deformation of the trunk, and abnormal urine flow [18]. Initially, hypospadias is usually diagnosed after birth during a physical examination of the newborn, when boys show ventral skin deficiency with a dorsal hood of the foreskin and an abnormally located opening with varying degrees of curvature of the penis [19]. A review conducted in the U.K. showed the posterior urethral valve incidence in patients with hypospadias was 4.5% of cases diagnosed with urethrocystoscopy [20].

In our study, to diagnose types of hypospadias, the following parameters were studied: the size of the glans penis, the shape of the navicular fossa, the degree of ventral deformation, curvature of the cavernous bodies, the location and size of the diameter of the dystopian meatus, the distance of the defect length between the glans and the meatus, and the type of urination. The age of the patient plays an important role in hypospadias treatment in boys. Urethroplasty in infants does not exclude the development of postoperative complications. According to some authors, an increased incidence of postoperative complications is observed in older children [19,21,22].

In our study, the average age of the patients was 48 months. At the present stage, hypospadias is corrected only surgically, depending on the form of hypospadias, using one- and two-stage operations, such as the MAGPI, Duplay, Snodgrass, Y–V Modified Mathieu, Duckett, Snodgraft, Hodgson III, Duckett, Faizulin VIII, Bracka, and Cukcow procedures. However, despite the many proposed operation methods, various numbers and types of complications are observed both in the early and late postoperative period [23,24,25,26,27]. In the group of children that underwent a procedure using the SMU I method, complications were observed only in the form of urethral fistula in 7.1% (1) of the patients, and in the remaining 92.9% (13 patients), this type of complication was absent. In subgroup A2, fistulas were observed in 17.9% (5) of the patients, and the remaining 82.1% (23) did not display fistulas. Traditional methods of treating hypospadias can lead to postoperative complications such as a long duration of wound healing, suture divergence, and urethral fistulas of the neomeatus.

In our study, in subgroup B2, urethral fistulas developed in 35.8% (19) of the patients after Snodgraft and Bracka surgery, and fistulas occurred in 25.0% (5 patients) after urethroplasty using the MAGPI method. The most common complications after Snodgrass surgery were urethral fistulas (40.5%) and meatal stenosis (11.1%). In some cases, urethral strictures and chordal recurrences were observed (3.2% and 2%, respectively) [28]. In order to reduce the development of complications after traditional urethroplasty, surgical methods have been proposed using the vaginal process of the testicle, the fleshy shell of the foreskin, the fascia of the penis trunk, and other various tissues as a protective layer for the neourethra [29,30,31,32].

For the treatment of the distal and midshaft types of hypospadias using the TIPU method, a flap of foreskin on the pedicle was used as a protective layer and for the skin grafting of the ventral surface of the trunk of the penis. In 8.1% of the cases, meatus stenosis (5.4%) and cutaneous fistulas (2.7%) were observed [33,34]. After a two-stage operation using a free flap of foreskin and cheek mucosa with a protective layer of the tunica dartos urethral suture, complications were noted: urethral fistulas in 7.1% of the patients, urethral stenosis in 7.1%, and graft contracture in 13% [35].

We proposed hypospadias correction using a vascularized flap of skin and the fleshy shell of the foreskin [36,37]. For the first time, Snow used the vaginal membrane to close the suture of an artificial urethra during the second stage of urethroplasty; since then, many authors have supplemented hypospadias correction and urinary fistula elimination using this method [38]. Urethroplasty with a dorsal free graft of the inner skin of the foreskin (DIGU, dorsal inlay graft urethroplasty) has been described as an additional method to classic tubular dissected plate (TIP) urethroplasty, which is aimed at reducing the risk of neourethral stenosis [39]. The results of the research showed the development of complications in the later postoperative period [19].

The results of using a free foreskin skin graft with a length of no more than 35 mm in the treatment of mid- and proximal shaft hypospadias showed the development of various types of complications which required repeated surgical intervention: meatus stenosis, urethral fistulas of various localization, urethral diverticula, and impaired urine flow [40]. The rate of postoperative complications such as urethral fistula decreased by 4.2% and amounted to 12.6%; the rate of neomeatus dystopia decreased by 100% and amounted to 0%. The proposed methods of distal and shaft hypospadias surgery in boys showed advantages in the duration of postoperative recovery and achievement of aesthetic appearance and functional condition in comparison with traditional methods. At the same time, we believe that long-term postoperative monitoring of this category of patients is necessary in order to promptly identify and eliminate late-stage complications.

## 5. Conclusions

The proposed new methods of distal and shaft hypospadias surgery, in comparison with traditional surgical techniques, have the following advantages: good cosmetic appearance of the penis, complete straightening of the shaft, and right outflow of urine through the slit-like neomeatus at the apex of the glans penis.

## Figures and Tables

**Table 1:** Comparability of the studied groups.

Characteristic		Frequency	Groups		Total	x^2^	*p*
			General Investigation (A1 and B1), n = 59	General Comparison (A2 and B2), n = 77			
Diagnosis	Glanular	n (%)	1 (33.3)	2 (66.7)	3 (100)	0.522	>0.05
	Coronal	n (%)	8 (57.1)	6 (42.9)	14 (100)		
	Distal-shaft	n (%)	14 (53.8)	12 (42.6)	26 (100)		
	Midshaft	n (%)	32 (39.0)	50 (61.0)	82 (100)		
	Proximal-shaft	n (%)	4 (36.4)	7 (63.6)	11 (100)		
	Total	n (%)	59 (43.4)	77 (56.6)	136 (100)		
Localization of meatus	Glans of penis	n (%)	1 (25.0)	3 (75.0)	4 (100)	0.573 *	>0.05
	Corona of penis	n (%)	8 (47.1)	9 (52.9)	17 (100)		
	Upper part of penis trunk	n (%)	19 (52.8)	17 (47.2)	36 (100)		
	Middle part of penis trunk	n (%)	27 (38.0)	44 (62.0)	71 (100)		
	Lower part of penis trunk	n (%)	4 (50)	4 (50)	8 (100)		
	Total	n (%)	59 (43.4)	77 (56.6)	136 (100)		
The degree of curvature of the cavernous bodies of the penis	Insignificant	n (%)	0	2 (100)	2 (100)	0.084 *	>0.05
	Mild	n (%)	8 (28.6)	20 (71.4)	28 (100)		
	Moderate	n (%)	46 (46.5)	53 (53.5)	99 (100)		
	Severe	n (%)	5 (71.4)	2 (28.6)	7 (100)		
	Total	n (%)	59 (43.4)	77 (56.6)	136 (100)		

* Fisher’s exact test was used to assess the significance level of differences between the two groups.

**Table 2:** Comparability of the studied groups by age.

Investigation Subgroups	Age (Months)			*p*
	Median	Q1	Q3	
General Investigation (A1 and B1)	49	41	72	0.641 *
General Comparison (A2 and B2)	59	41	72	

* *p* > 0.05.

**Table 3:** Comparability of subgroups of Group I by diagnosis.

Characteristic		Frequency	Group I, Subgroups A1 and A2		Total	x^2^	*p*
			SMU I	Snodgrass MAGPI			
	Glanular	n (%)	1 (50)	1 (50)	2 (100)	0.365 *	>0.05
	Coronal	n (%)	8 (66.7)	4 (33.3)	12 (100)		
	Distal-shaft form	n (%)	9 (52.9)	8 (47.1)	17 (100)		
	Midshaft	n (%)	8 (50)	8 (50)	16 (100)		
	Proximal-shaft form	n (%)	0	3 (100)	3 (100)		
	Total	n (%)	26 (52.0)	24 (48.0)	50 (100)		

* Fisher’s exact test was used to assess the significance level of differences between the two groups.

**Table 4:** Comparability of subgroups of Group I by clinical signs.

Characteristic		Frequency	Group I, Subgroups A1 and A2		Total	x^2^	*p*
			SMU I	Snodgrass MAGPI			
Localization of meatus	Glans of penis	n (%)	1 (50)	1 (50)	2 (100)	0.788 *	>0.05
	Corona of penis	n (%)	8 (61.5)	5 (38.5)	12 (100)		
	Upper part of penis trunk	n (%)	13 (52.0)	12 (48.0)	25 (100)		
	Middle part of penis trunk	n (%)	4 (40)	6 (60)	10 (100)		
	Total	n (%)	26 (52)	24 (48)	50 (100)		
The degree of curvature of the cavernous bodies of the penis	Insignificant	n (%)	0 (0)	1 (100)	1 (100)	0.064 *	>0.05
	Mild	n (%)	7 (33.3)	14 (66.7)	21 (100)		
	Moderate	n (%)	18 (66.7)	9 (33.3)	27 (100)		
	Severe	n (%)	1 (100)	0 (0)	1 (100)		
	Total	n (%)	26 (52)	24 (48)	50 (100)		
The direction of the urine stream	Straight	n (%)	0 (0)	1 (100)	1 (100)	0.163 *	>0.05
	To the right	n (%)	0 (0)	2 (100)	2 (100)		
	To the left	n (%)	2 (100)	0 (0)	2 (100)		
	Down	n (%)	24 (53.3)	21 (46.7)	45 (100)		
	Total	n (%)	26 (52)	24 (48)	50 (100)		

* Fisher’s exact test was used to assess the significance level of differences between the two groups.

**Table 5:** Comparability of subgroups of Group I by age.

Investigation Subgroups		Age (Months)			*p*
		Median	Q1	Q3	
Group I	Subgroup A1	48	30	120	0.733 *
	Subgroup A2	51	22	98	

* *p* > 0.05.

**Table 6:** Comparability of subgroups of Group II by diagnosis.

Characteristic		Frequency	Group II, Subgroups B1 and B2		Total	x^2^	*p*
			SMU II	Snodgraft, Bracka			
Diagnosis	Glanular	n (%)	0 (0)	1 (100)	1 (100)	0.469 *	>0.05
	Coronal	n (%)	0 (0)	2 (100)	2 (100)		
	Distal-shaft	n (%)	5 (55.6)	4 (44.4)	9 (100)		
	Midshaft	n (%)	24 (36.4)	42 (63.6)	66 (100)		
	Proximal-shaft types	n (%)	4 (50)	4 (50)	8 (100)		
	Total	n (%)	33 (38.4)	53 (61.6)	86 (100)		

* Fisher’s exact test was used to assess the significance level of differences between the two groups.

**Table 7:** Comparability of subgroups of Group II by clinical signs.

Characteristic		Frequency	Group II, Subgroups B1 and B2		Total	x^2^	*p*
			SMU II	Snodgraft Bracka			
Localization of meatus	Glans of penis	n (%)	0 (0)	1 (100)	1 (100)	0.247 *	>0.05
	Corona of glans	n (%)	0 (0)	4 (100)	4 (100)		
	Upper part of penis trunk	n (%)	6 (54.5)	5 (45.5)	11 (100)		
	Middle part of penis trunk	n (%)	23 (37.7)	38 (62.3)	66 (100)		
	Proximal part of penis trunk	n (%)	4 (50)	4 (50)	8 (100)		
	Total	n (%)	33 (38.4)	53 (61.6)	86 (100)		
The degree of curvature of the cavernous bodies of the penis	Insignificant	n (%)	0 (0)	1 (100)	1 (100)	0.223 *	>0.05
	Mild	n (%)	1 (14.3)	6 (85.7)	7 (100)		
	Moderate	n (%)	28 (38.9)	44 (61.1)	72 (100)		
	Severe	n (%)	4 (66.7)	2 (33.3)	6 (100)		
	Total	n (%)	33 (38.4)	53 (61.6)	86 (100)		
Direction of the urine stream	Straight	n (%)	0 (0)	2 (100)	2 (100)	0.064 *	>0.05
	To the right	n (%)	1 (16.7)	5 (83.3)	6 (100)		
	To the left	n (%)	3 (100)	0 (0)	3 (100)		
	Down	n (%)	29 (38.7)	46 (61.3)	75 (100)		
	Total	n (%)	33 (38.4)	53 (61.6)	86 (100)		

* Fisher’s exact test was used to assess the significance level of differences between the two groups.

**Table 8:** Comparability of subgroups of Group II by age.

Investigation Subgroups		Age (Month)			*p*
		Median	Q1	Q3	
Group II	Subgroup B1	56	12	192	0.947 *
	Subgroup B2	60	16	185	

* *p* > 0.05.

**Table 9:** Postoperative complications: study Group I and Group II.

Postoperative Complications		Frequency	Group I, Subgroups A1 and A2				Group II, Subgroups B1 and B2			
			SMU I	Snodgrass MAGPI	Total	*p*	SMU II	Snodgraft Braska	Total	*p*
Urethral fistula	No	n (%)	25 (56.8)	19 (43.2)	44 (100)	0.065	29 (87.9)	34 (64.2)	63 (73.3)	0.016 *
	Yes	n (%)	1 (16.7)	5 (83.3)	6 (100)		4 (12.1)	19 (35.8)	23 (26.7)	
	Total	n (%)	26 (52.0)	24 (48.0)	50 (100)		33 (100)	53 (100)	86 (100)	
Dystopia of neomeatus	No	n (%)	26 (100)	20 (83.3)	46 (92.0)	0.030 *	33 (100)	45 (84.9)	78 (90.7)	0.019 *
	Yes	n (%)	0 (0)	4 (16.7)	4 (8.0)		0 (0)	8 (15.1)	8 (9.3)	
	Total	n (%)	26 (100)	24 (100)	50 (100)		33 (100)	53 (100)	86 (100)	
Post surgical wound healing	Primary tension	n (%)	26 (100)	17 (70.8)	43 (86.0)	0.003 *	32 (97.0)	37 (69.8)	69 (80.2)	0.002 *
	Secondary tension	n (%)	0 (0)	7 (29.2)	7 (14.0)		1 (3.0)	16 (30.2)	17 (19.8)	
	Total	n (%)	26 (100)	24 (100)	50 (100)		33 (100)	53 (100)	86 (100)	

* *p* < 0.05.

## Data Availability

The data necessary to reproduce the results presented here are not publicly accessible, as the participants’ informed consent did not include public data sharing, but are available from the first author upon reasonable request.
